# Comparison and Enumeration of Chemical Graphs

**DOI:** 10.5936/csbj.201302004

**Published:** 2013-02-26

**Authors:** Tatsuya Akutsu, Hiroshi Nagamochi

**Affiliations:** a Bioinformatics Center, Institute for Chemical Research, Kyoto University, Gokasho, Uji, Kyoto 611-0011, Japan; b Graduate School of Informatics, Kyoto University, Yoshida, Kyoto 606-8501, Japan

**Keywords:** unique naming, maximum common subgraph, kernel methods, structural isomers, stereoisomers

## Abstract

Chemical compounds are usually represented as graph structured data in computers. In this review article, we overview several graph classes relevant to chemical compounds and the computational complexities of several fundamental problems for these graph classes. In particular, we consider the following problems: determining whether two chemical graphs are identical, determining whether one input chemical graph is a part of the other input chemical graph, finding a maximum common part of two input graphs, finding a reaction atom mapping, enumerating possible chemical graphs, and enumerating stereoisomers. We also discuss the relationship between the fifth problem and kernel functions for chemical compounds.

## Introduction

In computer analysis of chemical compounds, chemical structures are usually represented as graph structured data. Mathematically, a graph consists of a set of vertices and a set of edges, where a vertex represents some object and an edge represents a relation between two objects. From a chemical viewpoint, a graph corresponds to a chemical structural formula, in which a vertex and an edge correspond to an atom and a chemical bond, respectively. Furthermore, an atom type and a bond type are represented by labels of a vertex and an edge, respectively. In this article, graphs with such labels are called *chemical graphs*.

Graphs are a very important concept in computer science, and extensive studies have been done to develop efficient algorithms for a number of graph problems. Among these problems, we focus on fundamental problems relevant to chemical graphs. In particular, we consider comparison and enumeration of chemical graphs because these are fundamental and have a long history in chemoinformatics. For example, unique naming of chemical compounds and enumeration of isomers have been studied for more than 100 years [1], much before the invention of computers. In this article, we consider the following problems:

- determining whether two chemical graphs are identical,
- determining whether one input chemical graph is a part of the other input chemical graph,
- finding a maximum common part of two input graphs,
- finding a reaction atom mapping,
- enumerating possible chemical graphs,
- enumerating stereoisomers,

where (i) and (v) are closely related to unique naming and enumeration of structural isomers, respectively. We do not intend to provide a comprehensive review on these problems because there are too many methods even for any of these six problems. Instead, we try to clarify the *computational complexities* of them. We also introduce some recent developments on problems (v) and (vi) with focusing on our recent work because our algorithms are based on somewhat different approaches than traditional approaches in chemoinformatics [2] and they have guaranteed computational complexities. Since this review article focuses on time complexity aspects of the problems and algorithms, readers interested in practical and heuristic methods in chemoinformatics are referred to existing books and review articles: [2] for fundamental algorithms, [3, 4] for pattern matching algorithms, [2, 5, 6] for prediction and regression methods, and [2, 7] for enumeration algorithms.

As discussed later, most of the above problems are intractable for general graphs from a viewpoint of computational complexity. However, chemical graphs have several restrictions. For example, the maximum number of bonds connecting to an atom is usually less than 8. Making use of these constraints, it is often possible to develop theoretically efficient algorithms. Therefore, before discussing individual problems, we briefly review graph classes that are relevant to chemoinformatics.

The organization of this article is as follows. First, we review graph classes relevant to chemoinformatics and give a brief introduction of computational complexity. Next, we review theoretical results and some algorithms on problems (i)-(iv). Next, we describe a relationship between kernel methods and enumeration problems, where kernel methods are a kind of machine learning method and have been applied to various chemoinformatics problems. Then, we review our recent algorithms for problems (v) and (vi) because they are based on state-of-the-art techniques in graph algorithms and thus may bring new methodologies into chemoinformatics. Finally, we conclude with future work. For the purpose of simplicity of presentation, we do not give formal definitions, instead explain terms and results by using words and figures.

## Graphs and chemical compounds

### Chemical graphs

A *graph* consists of a set of *vertices* and a set of *edges*, where a vertex and an edge correspond to an atom and a chemical bond, respectively. Each graph is denoted as *G*(*V,E*) where *V* denotes a set of vertices and *E* denotes a set of edges. There are two kinds of graphs: *directed graphs* and *undirected graphs*. Each edge has a direction in directed graphs, whereas no edge has a direction in undirected graphs. Since there is usually no explicit direction in chemical bonds, we only consider undirected graphs in this article and thus each edge is represented by a set of two vertices (i.e., two atoms connected by the corresponding chemical bond).

In order to associate chemical structures to graphs, we employ *labels* of vertices and edges. Each vertex *v* has a label *l*(*v*), which represents an atom type (e.g., *l*(*v*) = ‘C’ if *v* corresponds to a carbon atom). In this article, a graph with vertex and edge labels defined as above is called a *chemical graph*. There are two ways to represent a chemical bond with multiplicity:

- multiplicity is represented by multi-edges (e.g., double bond is represented by two edges),
- multiplicity is represented by a label of an edge (e.g., *l*(*e*) = 2 if an edge *e* corresponds a double bond).

We mainly consider the latter way of representing chemical bonds in this article, where *l*(*e*) = 1.5 may represent an aromatic bond. The *degree* of a vertex is defined as the number of edges connecting to it, and is closely related to the valence of an atom. A graph with a designated vertex *r* is called a graph *rooted* at a vertex *r*. Isomorphism between two rooted graphs assumes that the roots of the two graphs correspond each other. In this paper, we utilize a fast algorithm designed for enumerating rooted trees. However, we designate as the root of a tree a special vertex of the tree which is uniquely determined by the topological structure only, and thereby our algorithms effectively enumerate ‘‘unrooted” trees.

### Graph classes

As mentioned above, chemical structures can be represented as graphs. However, we need not consider all kinds of graphs. For example, it is known that most atoms have valence at most 8, which implies that the maximum degree of chemical graphs is at most 8. Therefore, it is enough for chemical structures to consider graphs with bounded degree. In what follows, we only consider bounded degree graphs.

**Figure 1:**
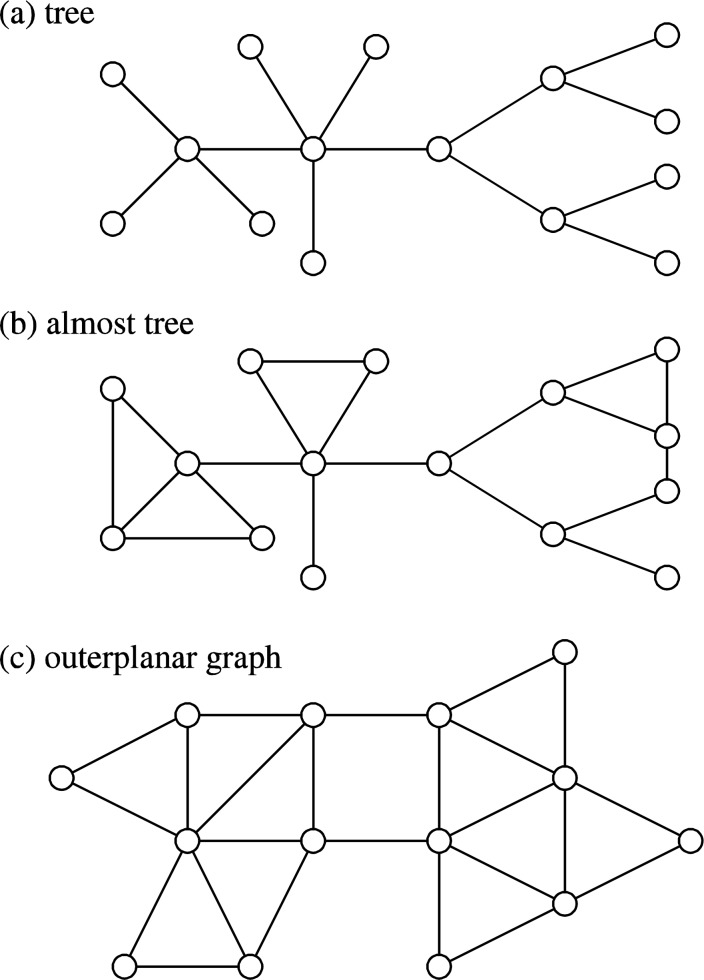
Examples of (a) tree, (b) almost tree, and (c) outerplanar graph, where *k*=2 in (b).

As discussed later, many graph problems can be solved much faster if we restrict types of graphs. Therefore, we review here several graph classes that are relevant to chemical applications (see Figure 1). For details of graph classes, see [8].

**Tree:** A graph is called a *tree* if it is connected and does not have a loop, where ‘connected’ means that there exists a path (a sequence of connected edges) connecting any pair of vertices. Trees are one of the simplest graphs and many problems can be solved much more efficiently for trees than for general graphs.

**Outerplanar graph:** A graph is called *outerplanar* if it can be drawn on a plane in such a way that all vertices lie on the outer face without crossing of edges, where the outer face is the unbounded exterior region. Trees are a subclass of outerplanar graphs.

**Almost tree:** A graph is called an *almost tree* (with parameter *k*) if each biconnected component (i.e., maximal non-tree part) is obtained by adding at most *k* edges to a tree. Trees are a subclass of almost trees (i.e., *k*=0). However, outerplanar graphs are not a subclass of almost trees or almost trees are not a subclass of outerplanar graphs.

**Figure 2:**
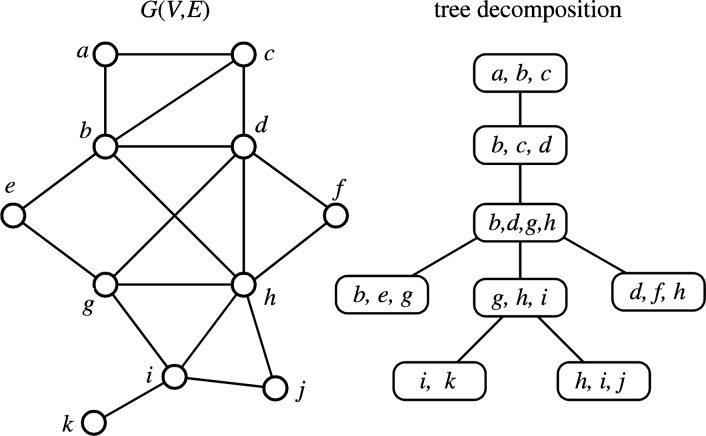
**Examples of partial** ***k*** **-tree for** ***k*** **=3**. The right figure shows a tree decomposition of *G*(*V*,*E*).

**Partial** ***k*** **-tree:** A graph is called a *partial k-tree* if it is transformed into a tree by regarding a family of subsets of vertices as a set of new vertices (i.e., by tree decomposition), where each subset consists of at most *k*+1 vertices (Figure 2). Trees, outerplanar graphs, and almost trees with parameter *k* are subclasses of partial 1-trees, partial 2-trees, and partial *k*+1-trees, respectively [9].

Yamaguchi et al. studied the distribution of partial *k*-trees in chemical graphs [10]. Horváth and Ramon also studied the distribution of partial *k*-trees in some dataset and reported that 8.77%, 97.35% and 99.97% of compounds are partial 1-trees, 2-trees, and 3-trees, respectively and most partial 2-tree compounds are outerplanar [11].

### Computational complexity

Here, we briefly review some basic concepts in computational complexity. If the computation time of an algorithm is proportional to *n* ^*d*^ (i.e., the computation time is *O*(*n* ^*d*^)) for some constant *d* where *n* denotes the size of an input data, the algorithm is said to be a *polynomial-time algorithm*. There exist many problems that do not have polynomial-time algorithms. Although we do not explain details, *NP-hard* problems are widely believed not to have polynomial-time algorithms. In theoretical computer science, polynomial-time algorithms are regarded as efficient algorithms whereas NP-hard problems are regarded as intractable problems. However, NP-hardness does not necessarily mean practical inefficiency. In particular, the number of vertices in a chemical graph is usually less than 100. Therefore, there is room for development of practically efficient algorithms for chemical compounds even if the problems are NP-hard.

## Comparison of chemical graphs

**Figure 3:**
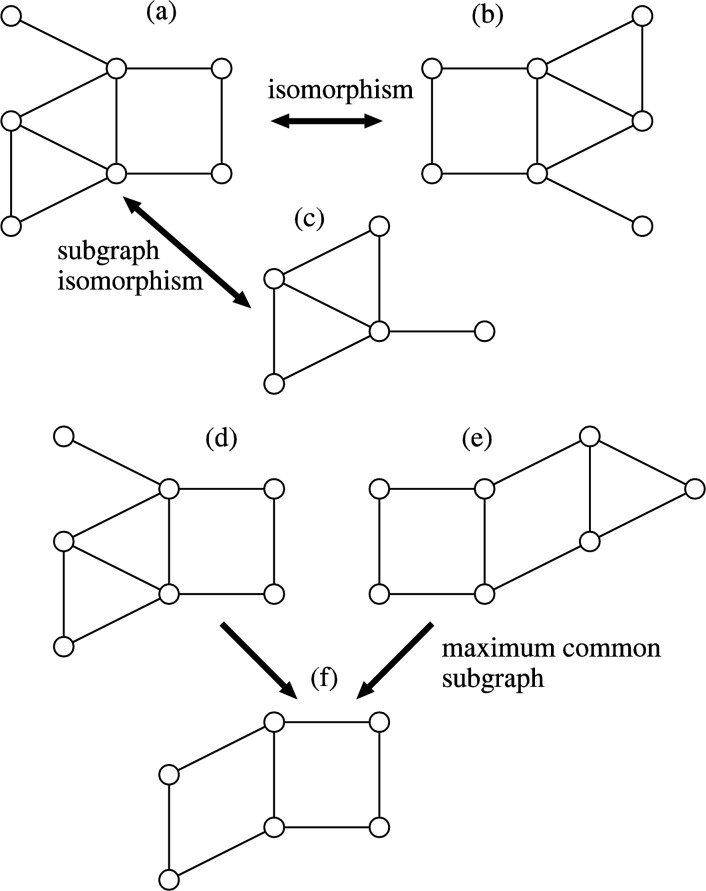
**Comparison of graphs**. Graph (a) is isomorphic to graph (b). Graph (c) is subgraph isomorphic to graph (a). Graph (f) is a maximum common subgraph between graphs (d) and (e).

**Figure 4:**
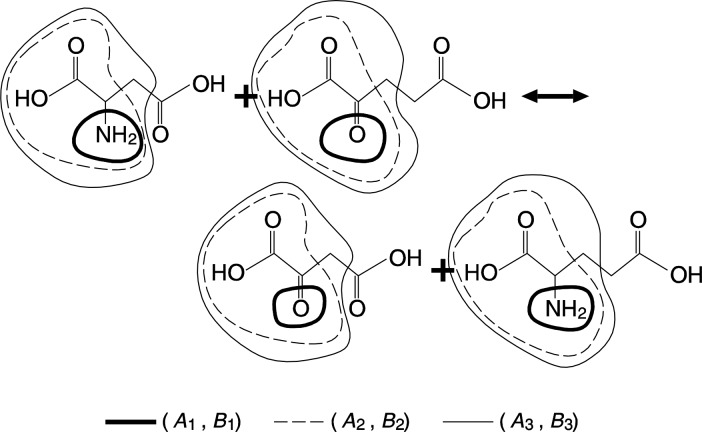
**Reaction atom mapping**. In this case, there exist three possible reaction atom mappings, where (*A*_1_,*B*_1_) is the most plausible. By deleting the edges (bonds) crossing to curves of each type, we can obtain isomorphic graphs between the left hand side and right hand side of the reaction, which also gives a sequence of graph editing operations.

Comparison of graph structured data is fundamental and important for chemoinformatics and pattern recognition. Indeed, extensive studies have been done to develop practical algorithms for that purpose [2–4]. In computer science, extensive theoretical studies have also been done for comparison of graphs. However, it seems that these theoretical results are not well-known in chemoinformatics. Although most of theoretical algorithms are not efficient in practice, they might give some hints for development of practically efficient algorithms. In this section, we mainly review theoretical results on graph isomorphism, subgraph isomorphism, maximum common subgraphs, and reaction atom mapping, all for chemical graphs (Figure 3 and Figure 4).

### Isomorphism and signature

The most fundamental problem for comparison of chemical graphs is to test whether given two graphs are identical (i.e., *isomorphic*). Although it is unknown whether there exists a polynomial-time algorithm for general graphs, a polynomial-time algorithm is known for graphs of bounded degree [12], which means that isomorphism of two chemical graphs can be tested in polynomial time. However, this algorithm is not practical because it is based on group theory and the degree of polynomial in the time complexity is high.

It is also important to give a *normal form* of a given graph, which is equivalent to giving a unique name to a graph. Indeed, huge efforts have been paid in chemistry to define rules for giving unique names to chemical structures (e.g., IUPAC nomenclature). In computer science, a group-theoretic polynomial-time algorithm is also known for the normal form problem for graphs of bounded degree [13] although it is not practical. Faulon gave detailed discussions on isomorphism and normal forms for chemical graphs and presented polynomial-time algorithms for chemical graphs with planar structures [14].

### Subgraph isomorphism

Another important problem is to decide whether some chemical graph is included as a part of another chemical graph. Deciding whether a benzene ring is contained in a given chemical graph is an example of this problem. This problem is called the *subgraph isomorphism* problem in graph theory. Although it is known that the subgraph isomorphism problem is NP-hard even for graphs of the maximum degree 3, a polynomial-time algorithm is known for partial *k*-trees of bounded degree [15, 16]. Therefore, the subgraph isomorphism problem can be solved in polynomial time for almost all chemical graphs. Unfortunately, the algorithms in [15, 16] are not practical.

For practical applications to chemical compounds, subgraph isomorphim algorithms based on maximum clique or a branch-and-bound method [17] have been widely developed and utilized (see for example, [18] for the former approach and [19] for the latter one). Although they are not guaranteed to work in polynomial time, they work fast in practice. It is also important to search graphs containing subgraphs isomorphic to a given query graph in a chemical database [18, 19]. In such an application, filtering non-relevant compounds is quite useful and thus various features have been proposed and utilized (see for example, [19]).

### Maximum common subgraph

It is also important to find a common part of two or more chemical graphs. Among several possible formulations, the most fundamental one is the maximum common subgraph problem (precisely, the maximum common connected edge subgraph problem) for two graphs [3]. Since a polynomial-time algorithm was developed for almost trees of bounded degree [20], there had been almost no significant progress [10] until recently from a viewpoint of the computational complexity. However, Akutsu and Tamura recently developed a polynomial-time algorithm for outerplanar graphs of bounded degree [21]. On the other hand, they also showed that the problem remains NP-hard for partial *k*-trees of bounded degree with *k* =11 [22].

For general graphs, some exponential time algorithms have been developed. Huang et al. presented an *O*(*n* ^*h*^ *h* ^2^) time algorithm and showed an *O*(*f*(*h*)*n* ^*Ω*(*h*)^) time lower bound under some reasonable assumption on complexity class where *n* is the number of vertices of a larger input graph, *h* is the size of the maximum common subtree, and *f* is any recursive function [23]. However, we could not confirm *O*(*n* ^*h*^ *h* ^2^) time complexity and speculate that it should be *O*(*n* ^*2h*^ *h* ^2^). Abu-Khzam et al. developed an *O*(1.274^*c*^+3^*n*/3^(*n*+1)^*c*^) time algorithm where *c* is the size of the minimum vertex cover of a smaller input graph [24].

### Reaction atom mapping

In addition to comparison of chemical graphs, there exists another important pattern matching problem for graphs: finding a minimum cost sequence of graph editing operations that transforms one input graph into the other input graph [25]. There exist several variants of the problem depending on the definition of editing operations [26]. There also exists a close relationship between the minimum graph edit problem and the maximum common subgraph problem, both of which are known to be NP-hard in general [25]. Since minimum graph edit is too wide, we focus on the *reaction atom mapping* problem (Figure 4). It is a problem of finding an optimum correspondence (or enumerating all possible mappings) between atoms before and after a reaction. It is also defined as a problem of finding a minimum cost sequence of deletions and additions of edges that transforms one input graph into the other input graph where input graphs can be disconnected and the set of atoms must be preserved before and after the edit sequence. Consider a simple reaction of the following type

X-A + Y-B ⟷ X-B + Y-A

where X, Y, A, B are chemical species. Then, by deleting two edges (edges between X and A, and between Y and B) from the left hand side of the reaction and inserting two edges (edges between X and B, and between Y and A), we have the right hand side of the reaction. It also gives a mapping between atoms before and after the reaction. However, mapping or edit sequence may not be determined uniquely as shown in Figure 4. In such a case, it may be required to enumerate all possible mappings, or to find a chemically optimal mapping. Since there exist several formulations depending on applications, we do not give precise definitions here. This problem has several applications including consistency check of chemical reactions in a database and in silico tracer experiments.

Arita developed a heuristic method for the reaction atom mapping problem based on maximum common subgraph (MCS) [27]. Hattoti et al. also applied their own maximum common subgraph (MCS) algorithm to find reaction atom mappings [28]. However, Arita pointed out that MCS approaches sometimes fail to find desired mappings [27]. Akutsu firstly gave a mathematical formalization of the problem, proved NP-hardness of the problem, and developed an algorithm based on unique graph naming and exhaustive examination of cutting edges [29]. Crabtree and Mehta developed faster algorithms based on unique graph naming and efficient combinatorial search for cutting edges [30]. Heinonen et al. also developed a fast algorithm based on A* search [31], a well-known efficient searching technique in artificial intelligence. Zhou and Nakhleh developed a method to find all symmetries in both a chemical compound and a chemical reaction, where the latter can be used to find reaction atom mappings with stereo chemical information [32].

## Kernel methods and pre-image problem

Recently, kernel methods, which include support vector machines (SVMs), have become one of the standard tools in machine learning. Kernel methods have also been extensively applied to Quantitative Structure-Activity Relationship (QSAR) and Quantitative Structure-Property Relationship (QSPR) problems [2, 5, 6] whose purposes are to predict the chemical activity and property for a given chemical compound, respectively. For example, various kernel functions for QSAR/QSPR have been developed based on alignment of two chemical graphs [33, 34], three-dimensional superposition [35], Tanimoto and other coefficients [36], molecular descriptors [37], and subtree patterns [38].

In order to apply kernel methods to chemical structures, it is usually required to map a chemical graph to a feature vector in a feature space (i.e., a vector in high-dimensional Euclidean space or infinite-dimensional Hilbert space) because a kernel function is defined as an inner-product between two feature vectors. Although various methods have been proposed for design of feature vectors for chemical graphs, those based on *frequency of small fragments* [39–41] and *frequency of labeled paths* [42, 43] have been widely used, where weights/probabilities are sometimes put on paths/fragments.

**Figure 5:**
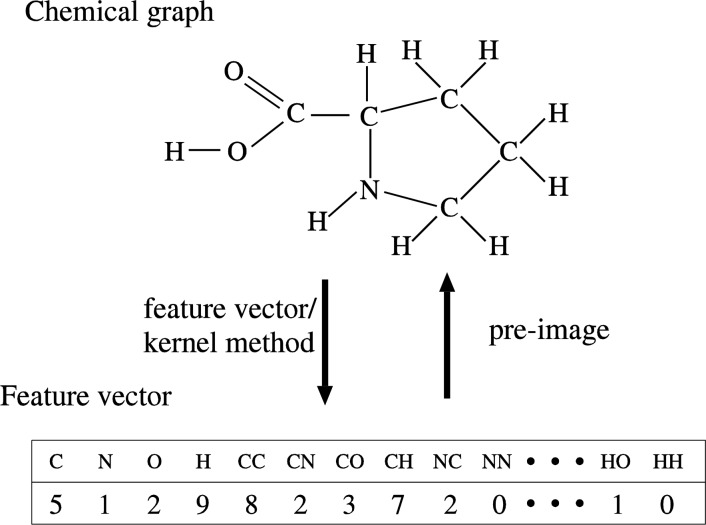
Feature vector and pre-image.

For example, consider chemical compounds consisting of atoms of type C, N, O, H. Then, there are 4 kinds of labeled paths of length 0 (i.e., C, N, O, H), 16 kinds of labeled paths of length 1 (i.e., C-C, C-N,...), 64 kinds of labeled paths of length 2, and so on. Figure 5 shows an example of a feature vector based on frequency of labeled paths (precisely, a feature vector based on the numbers of occurrences of labeled paths). For a chemical graph shown in Figure 5, the numbers of atoms of type C, N, O, H are 5, 1, 2, 9, respectively, and thus the coordinate values corresponding to paths of length 0 are 5, 1, 2, 9. For the same graph, the number of C-N bonds is 2 and thus the corresponding coordinate value is 2. For C-C bond, the coordinate value is 8 because each C-C bond is counted twice as two paths for opposite directions. The maximum length of paths of a feature vector is called the *level* of the feature vector. Therefore, the vector in Figure 5 is a feature vector of level 1.

In chemoinformatics, inverse QSAR/QSPR is becoming important because it might lead to design of new chemical compounds and new drugs [44–46]. As closely related concept, the pre-image problem has been studied in machine learning [47, 48]. In the pre-image problem, a desired object is specified or computed as a vector in a feature space using a suitable objective function and then the vector is mapped back to the input space, where this mapped back object is called a *pre-image*. Then, it is expected that this pre-image has a desired property (e.g., chemical activity). Akutsu et al. showed that the graph pre-image problem based on path frequency is solved in polynomial time of the number of atoms if the graphs are trees whose maximum degree is bounded by a constant and the lengths of given paths and the number of atom types are bounded by constants [49], which was further extended to outerplanar graphs with some constraints [50]. However, this algorithm is not practical due to its high degree of polynomial. Nagamochi proved that the graph pre-image problem can be solved in polynomial time for both tree and general graphs if the level of feature vectors is 1 [51]. Although the corresponding algorithm is efficient in practice, it can only be directly applied to feature vectors of level 1. Furthermore, it is difficult for the above mentioned algorithms to cope with various constraints. Therefore, it is useful to develop algorithms for the pre-image problem based on enumeration of chemical graphs, which is explained in the next section (see [52] for more detailed review).

It is to be noted that extensive studies have been done on inverse QSAR/QSPR, where the descriptors correspond to feature vectors in the pre-image problem. Kier et al. developed methods for reconstructing molecular structures from the count of paths of a length up to two and three by combining enumeration with bounding operations [44]. Skvortsova et al. developed a similar method where paths of the same length are further classified into several classes sing atom and bond information [45]. Faulon et al. defined a descriptor based on trees and developed methods to enumerate all the structures consistent with a given descriptor [46]. Wale et al. compared various descriptors including ECFP descriptors, not for inverse QSAR/QSPR but for compound retrieval and classification [53].

## Enumeration of chemical graphs and stereoisomers

The enumeration of chemical structures has a long history beginning from the work by Cayley in the 19th century [1]. Enumeration of chemical structures has many applications in chemistry, which include structure determination using mass-spectrum and/or NMR-spectrum, virtual exploration of chemical universe, reconstruction of molecular structures from their signatures, and classification of chemical compounds.

Some useful tools such as MOLGEN have been developed for enumeration of chemical graphs [2, 54]. Although MOLGEN is very efficient, it is worthy to examine other approaches because extensive studies have been done on graph enumeration in the field of theoretical computer science and data mining [55, 56]. In particular, it might be possible to develop faster algorithms for the enumeration and pre-image problems if we restrict the class of target chemical graphs and employ state-of-the-art techniques for enumeration of graph structures. Based on this idea, we have been developing several algorithms for enumeration of chemical graphs (i.e., structural isomers) and stereoisomers. Although our enumeration algorithms for structural isomers may not yet be faster in practice than MOLGEN, our algorithms have some guaranteed time complexities. In addition, our enumeration algorithms for stereoisomers are quite fast for counting: they work in optimal linear time and are very fast in practice. It is to be noted that the number of isomers grows exponentially to the number of atoms in general and thus it is impossible to output all isomers in polynomial time. Therefore, the purpose of development of fast enumeration algorithms is to reduce the computational complexity required per isomer. In this section, we explain key ideas used in these algorithms. Since some details are a bit involved, readers not interested in algorithmic details can skip such parts.

### Enumeration of chemical graphs

Given a feature vector *f* that specifies the frequency of each path of length at most *K*≥0, our aim is to enumerate all tree-like chemical graphs whose path frequency for all paths of length up to *K* is identical with *f*. A chemical graph is called *tree-like* if it becomes a simple tree (an acyclic graph) by replacing the multiple edges (bonds) between every two vertices with a single edge.

Ishida et al. [57] developed an efficient algorithm for enumerating all tree-like chemical graphs that satisfy a given single feature vector *f*. The algorithm consists of two major phases, each of which is designed based on a *branch-and-bound method* (see [17] for details), one of the standard enumerative approaches for solving combinatorial problems. In the first phase, we first design a branching operation to generate all simple trees that satisfy only the frequency of path of length 0 in the vector *f* (i.e., a set *V* of vertices is specified by *f*), ignoring any multiplicity prescribed in *f*. Starting with the empty graph, the operation appends a new vertex to the current tree until the tree has the vertex set *V*. A tree *T’* obtained from the current tree *T* by appending a new vertex is called a *child* of *T*, where *T* is called the *parent* of *T’*. Since the current tree *T* may have more than one adequate position to which a new vertex is appended (i.e., *T* may have more than one child), the parent-child relationship forms a tree rooted at the empty graph, called a *family tree*. Each node *v* with depth *k* in the family tree represents a tree *T*(*v*) of *k* vertices, and the trees of the descendants of *v*will be generated from *T*(*v*) by repeated applications of the branching operation.

**Figure 6:**
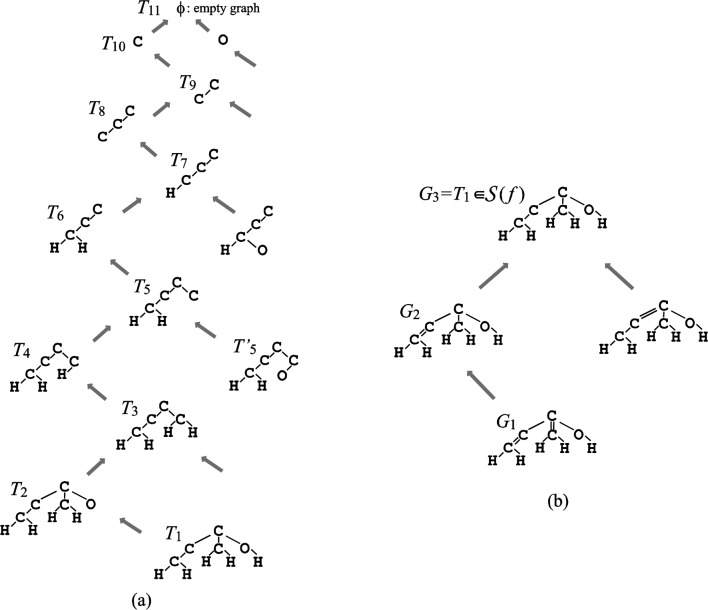
**Family trees for enumeration of chemical graphs**. (a) A family tree for generating simple trees; (b) A family tree for assigning multiplicity to simple tree *T*_1_∈*S*(*f*).

Figure 6 (a) shows part of a family tree for generating simple trees with a specified vertex set *V* of four carbons, one oxygen and five hydrogens, where the parent of each simple tree *T*_*i*_ is *T*_*i*+1_. A family tree is determined by how to define parents of trees, which also defines the children and affects the computational efficiency. Using the fastest tree enumeration [58], we can generate a child from the current tree in a constant time.

The branching operation is rather a straightforward enumeration which can output each tree efficiently, but may generate trees that do not satisfy some of the required conditions of the problem such as the frequency of path of length ≥ 1. In a branch-and-bound method, we also incorporate into the process of executing a branching operation, another procedure, called a *bounding operation*, to discard part of the process which produces only candidates that do not lead to any solutions. In our problem, we apply several quick tests to each node *v* in the family tree to try to know whether there is a descendant *w* of *v* such that *T*(*w*) is a solution; i.e., the tree *T*(*v*) can be extended to a simple tree on the specified vertex set *V* without violating any of the required conditions. Our bounding operation tests the following criteria and skips the process of appending vertices to *T*(*v*) if one of them holds:

1. The root of *T*(*v*) cannot be the centroid after any extension of *T*(*v*) (the centroid constraint);
2. The frequency of some path in *T*(*v*) exceeds the value specified by *f* (the feature vector constraint);
3. The valence of a labeled vertex in *T*(*v*) exceeds the valence of the atom (the valence constraint); and
4. *T*(*v*) cannot be extended to a tree with the vertex set *V* and edge set *E* specified by *f* (the detachment constraint). Whether criterion (4) holds or not can be tested efficiently by the algorithms in [51].

The first phase obtains a set *S*(*f*) of simple trees that satisfy the given path frequency ignoring the multiplicity. In the second phase, we select each simple tree *T*∈*S*(*f*) and generate all tree-like chemical graphs that satisfy the given feature vector *f* by assigning multiplicity on adequate edges in *T*. Our algorithm for the second phase is also designed based on a branch-and-bound method and the idea of family trees. Figure 6 (b) shows part of a family tree for assigning multiplicity to simple tree *T*_1_, where the parent of each tree-like graph *G*_*i*_ is *G*_*i*+1_.

Note that a feature vector *f* on path frequency specifies the exact number of times each path appears in a graph to be constructed. When a vector *f* is artificially constructed, no graph with such a path frequency in *f* may exist in many cases. To avoid this, Shimizu et al. [59] recently introduced a problem of constructing all tree-like graphs which satisfy one in a given set *F* of feature vectors. A set *F* of feature vectors is specified by a pair of upper and lower vectors *f*_*U*_ and *f*_*L*_ on path frequency such that *f*_*U*_ and *f*_*L*_ have the same frequency of path of length 0 (i.e, both specifies the same set of vertices), and the set *F* is defined to be the set of all vectors *f* such that *f*_*L*_≤*f*≤*f*_*U*_. Shimizu et al. [59] successfully designed a two-phase algorithm for handling the new problem directly without repeatedly applying the algorithm by Ishida et al. [57] to each feature vector *f*∈*F*.

Recently Suzuki et al. [60] have developed an algorithm for enumerating graphs with at most one cycle (of length at least 3) from a set *F* of feature vectors specified by upper and lower vectors *f*_*U*_ and *f*_*L*_ on path frequency. The main idea of this algorithm is to define the parent of a graph with exactly one cycle to be a tree-like graph by removing an edge in the cycle and to design as the third phase a procedure for generating a graph with exactly one cycle from each tree-like graph *T* obtained after the second phase of the algorithm by Shimizu et al. [59]. Our experimental result reveals that the computational efficiency of the new algorithm remains high considering the hardness of treating graphs with one cycle compared with tree-like graphs.

The computational efficiency of the above algorithms relies on the result that all vertex-colored trees with at most *n* vertices in constant time per output [58]. As an extension, we have proved that all trees with labeled vertices with exactly *n* vertices (resp., all rooted outerplanar vertex-labeled graphs with at most *n* vertices) can be generated in constant time per output [61] (resp., [62]).

### Enumeration of stereoisomers

**Figure 7:**
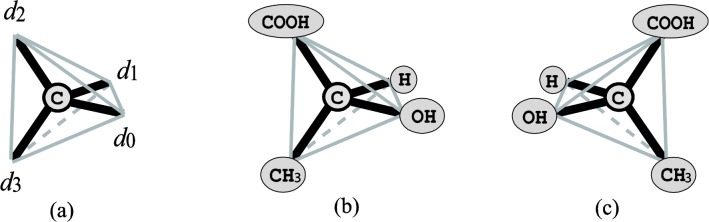
**Example of stereoisomers**. (a) The four directions *d*_0_, *d*_1_, *d*_2_ and *d*_3_ around a carbon atom in the three-dimensional space; (b), (c) Two configurations around the asymmetric carbon atom in lactic acid.

We have developed algorithms for enumerating all stereoisomers of a given chemical graph *G* that admits tree or outerplanar structures [63, 64]. The algorithms are based on *dynamic programming*, another standard enumerative approach for solving combinatorial problems (see [17] for details). For simplicity, we describe the idea only for tree-like graphs and the asymmetry around carbon atoms with no double bonds incident to them (the asymmetry formed by double bonds such as cis-trans type can be treated with a modified argument). Thus a carbon atom is *asymmetric* if the four substructures around it are all sterically distinct. Figure 7 (a) illustrates that the three-dimensional structure around a carbon atom forms a regular tetrahedron, where *d*_0_, *d*_1_, *d*_2_ and *d*_3_ represent the directions along the four edges incident to the carbon atom. Figure 7 (b) and (c) show two configurations around the asymmetric carbon atom in lactic acid.

**Figure 8:**
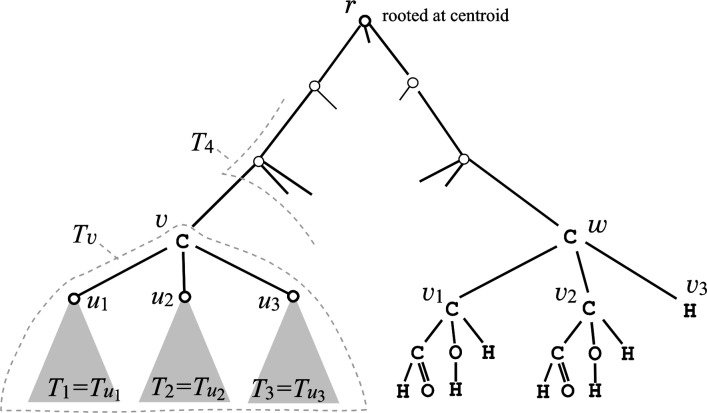
A tree-like chemical graph *G* rooted at its centroid *r*.

Given a tree-like chemical graph *G*, we regard it as a tree rooted at its centroid *r* (the vertex removal of which leaves no component containing more than a half number of the vertices). For each vertex *v* in *G*, we denote by *T*_*v*_ the subtree induced by *v* and all its descendants in *G*, and let *f*(*v*) denote the number of stereoisomers of *T*_*v*_. For a non-root carbon atom *v* in *G*, a stereoisomer of *T*_*v*_ is determined as follows. Let *u*_1_, *u*_2_ and *u*_3_ be the three children of *v* in *G*, where *v* is adjacent to the three subtrees $T_{i}=T_{u_{i}}$, *i*=1,2,3. Note that *v* is adjacent to the fourth subtree *T*_4_ composed of the rest of vertices, which is always structurally distinct from every $T_{i}=T_{u_{i}}$. See the carbon atom *v* in Figure 8 for four subtrees *T*_1_, *T*_2_, *T*_3_ and *T*_4_. Suppose that a stereoisomer of *T*_*v*_, say, the *k*-th one $T_{v}^{\left(k\right)}$, consists of the *k*_*i*_-th stereoisomer $T_{i}^{\left(k_{i}\right)}$ of *T*_*i*_ for each *i*=1,2,3, where *k*_*i*_∈{1,2,…,*f*(*u*_*i*_)}. Then we have two cases: (a) some two of $T_{1}^{\left(k_{1}\right)}$, $T_{2}^{\left(k_{2}\right)}$ and $T_{3}^{\left(k_{3}\right)}$ are sterically same (i.e., the same stereoisomer); and (b) every two of $T_{1}^{\left(k_{1}\right)}$, $T_{2}^{\left(k_{2}\right)}$ and $T_{3}^{\left(k_{3}\right)}$ are sterically distinct. In (b), *v* is asymmetric, and there are exactly two different three-dimensional positions of $\tau =\left\{T_{1}^{\left(k_{1}\right)},T_{2}^{\left(k_{2}\right)},T_{3}^{\left(k_{3}\right)}\right\}$ around *v*, each of which we denote by *σ*=+ or *σ*=-. In (a) *v* is symmetric, and we let *σ*=0. Thus, a stereoisomer $T_{v}^{\left(k\right)}$ of *T*_*v*_ is represented by (*τ,σ*) (or symbolically by an *index vector* (*k*_1_,*k*_2_,*k*_3_,*σ*)). Let *g*(*v*) (resp., *h*(*v*)) denote the number of collections *τ* of three stereoisomers of T_i_ by which case (a) occurs (resp., (b) can occur). Then the number of stereoisomers of *T*_*v*_ is given as *f*(*v*) = *g*(*v*) + 2*h*(*v*).

Our algorithm consists of two major phases: (i) the first phase counts the number *K*_*G*_ of stereoisomers of *G* by dynamic programming; and (ii) for each *k*=1,2,…,*K*_*G*_, the second generates the *k*-th stereoisomer by backtracking the computation in (i).

**Counting phase**. The first phase computes *f*(*v*) of each vertex *v* in a bottom-up manner along *G*. When we visit a non-root carbon atom *v*, *f*(*u*_*i*_) of each child *u*_*i*_, *i*=1,2,3 of *v* has been computed. If *T*_1_ and *T*_2_ are structurally same (where *f*(*u*_1_) = *f*(*u*_2_)) and *T*_3_ is structurally distinct from *T*_1_ and *T*_2_, then we have *g*(*v*) = *f*(*u*_1_)*f*(*u*_3_), $h(v)=\left(\begin{array}{c} f(u_{1})\\ 2 \end{array}\right)f(u_{3})$ and $f(v)=g(v)+2h(v)=f(u_{1})f(u_{3})+2\left(\begin{array}{c} f(u_{1})\\ 2 \end{array}\right)f(u_{3})$. Similarly if *T*_1_, *T*_2_ and *T*_3_ are all structurally distinct (resp., same), then we have *g*(*v*) = 0 and *h*(*v*) = *f*(*u*_1_)*f*(*u*_2_)*f*(*u*_3_) (resp., *g*(*v*) = *f*(*u*_1_)*f*(*u*_1_) and $h(v)=\left(\begin{array}{c} f(u_{1})\\ 3 \end{array}\right)$). For example, the carbon atom *v*_1_ in Figure 8 has no two children which have the structurally same subtree, and we have *g*(*v*_1_) = 0, *h*(*v*_1_) = 1 and *f*(*v*_1_) = 2. Note that *f*(*v*_2_) = 2 and *f*(*v*_3_) = 1. For the carbon atom *w* in Figure 8, only *v*_1_ and *v*_2_ among its children have the structurally same subtree, and we have *g*(*w*) = *f*(*v*_1_)*f*(*v*_3_) = 2, $h(w)=\left(\begin{array}{c} f(v_{1})\\ 2 \end{array}\right)f(v_{3})=1$ and *f*(*w*) = 4. We can regard the *f*(*w*) = 4 stereoisomers are represented by index vectors $T_{w}^{\left(1\right)}=(1,1,0)$, $T_{w}^{\left(2\right)}=(2,2,0)$, $T_{w}^{\left(3\right)}=(1,2,+)$, and $T_{w}^{\left(4\right)}=(1,2,-)$. By computing *f*(*v*) for all carbon atoms *v* in a bottom-up manner along *G*, we can finally determine the number *K*_*G*_ of all stereoisomers of *G*. The first phase can be implemented to run in *O*(*n*) time and space for a tree-like graph or outerplanar graph *G* with *n* vertices.

**Output phase**. For each number *i*=1,2,…,*K*_*G*_, the second phase outputs the *i*-th stereoisomer of *G*. The *i*-th stereoisomer of *G* will be characterized by choosing an index vector (*k*_1_,*k*_2_,*k*_3_,*σ*) for each carbon atom in a top-down manner along tree *G*. When we visit a carbon atom *v*, we are supposed to compute the *k*-th stereoisomer $T_{v}^{\left(k\right)}$ of the subtree *T*_*v*_ for a number *k*∈{1,2,…,*f*(*v*)} which has been determined by the process applied to the ancestors of *v* so far. Our task is to compute (*k*_1_,*k*_2_,*k*_3_,*σ*) from a given number *k*∈{1,2,…,*f*(*v*)}. For example, the carbon atom *w* in Figure 8 has *f*(*w*) = 4 stereoisomers, which are supposed to be represented by the index vectors in the above. Then if *k*=3 at *w*, then we take $T_{w}^{\left(3\right)}=(1,2,+)$ and assign *k*_1_=1 and *k*_2_=2 to our process for the children *v*_1_ and *v*_2_, respectively. Based on how *g*(*v*) and *h*(*v*) have been computed in the first phase, we can identify the *k*-th index vector without explicitly constructing a complete ranking table for the *f*(*v*) index vectors. We repeat this process until the corresponding index vector for each carbon atom in *G* is determined. The second phase can be implemented to generate each stereoisomer of a tree-like graph (resp., outerplanar graph) *G* with *n* vertices in *O*(*n*) (resp., *O*(*n* ^3^)) time per output using *O*(*n*) space.

## Summary and outlook

In this article, we have reviewed graph classes relevant to chemical compounds, theoretical results on the computational complexities of the isomorphism, normal forms, subgraph isomorphism, and maximum common subgraph problems of chemical graphs, and algorithms for computation of reaction atom mappings and enumeration of chemical graphs and stereoisomers.

As discussed above, the isomorphism and normal form problems can be solved in polynomial time for all chemical graphs and the subgraph isomorphism can be solved in polynomial time for almost all chemical graphs. However, these polynomial-time algorithms are not practical because of their high degrees of polynomials. Therefore, algorithms having low-degree polynomial complexities should be developed. As for the maximum common subgraph problem, there exists a polynomial time algorithm for chemical graphs with outerplanar structures, whereas it is NP-hard for partial *k*-trees with *k*=11. Since outerplanar graphs are a subclass of partial 2-trees and most chemical graphs are partial 3-trees, it is interesting to study the complexity of the maximum common subgraph problem for partial 2-trees and partial 3-trees. For the reaction atom mapping problem, several practical algorithms have been developed although it is NP-hard in general.

From a practical viewpoint, it is not necessary to develop polynomial-time algorithms because the size of chemical graphs is usually limited. Therefore, development of efficient exponential-time algorithms and/or fixed-parameter algorithms [65] is another theoretical approach to these problems. Of course, some of existing practical algorithms work very efficiently for most chemical graphs. Analysis of the computational complexities of such algorithms might also lead to development of faster algorithms.

In the latter part of this article, we explained algorithms for enumeration of chemical graphs and stereoisomers that were developed by the authors and their colleagues. These algorithms are fast in both theory and practice. They were implemented on the EnuMol web server (http://sunflower.kuicr.kyoto-u.ac.jp/tools/enumol2/) and are freely available via the web page for academic purposes, where the details of EnuMol will be reported elsewhere. However, the classes covered by these algorithms are limited. Therefore, development of algorithms that cover most chemical graphs is important future work. Of course, enumeration takes quite a long time for large chemical graphs because the number of objects to be enumerated grows exponentially to the number of atoms. Therefore, introduction and effective use of adequate constraints are important and necessary in future studies.
